# Tractional Retinal Detachment in a Patient With Waldenström’s Macroglobulinemia

**DOI:** 10.7759/cureus.12430

**Published:** 2021-01-02

**Authors:** Anastasia Tsiogka, Aristotelis Karamaounas, Evangelia Papakonstantinou, Petros Petrou

**Affiliations:** 1 Ophthalmology, 401 General Military Hospital, Athens, GRC; 2 Ophthalmology, "G. Gennimatas" General Hospital of Athens, Athens, GRC

**Keywords:** acute retinal necrosis, epiretinal membrane, tractional retinal detachment

## Abstract

An 86-year-old man with Waldenström’s macroglobulinemia and a history of acute retinal necrosis (four years ago) presented with reduced visual acuity in his right eye of three days' duration. He developed epiretinal membrane and tractional retinal detachment, which were treated successfully. Epiretinal membrane and tractional retinal detachment usually occur one to two months after the onset of retinitis. In our case, this happened four years after the diagnosis of acute retinal necrosis syndrome, prophylactic vitrectomy, and successful treatment of acute retinal necrosis syndrome in a patient with Waldenström’s macroglobulinemia.

## Introduction

It is commonly known that patients with cancer have an increased risk of opportunistic infections due to an immune dysfunction, which may be caused by antineoplastic chemotherapy, an underlying malignancy, or invasive procedures performed during supportive care [1]. Waldenström’s macroglobulinemia (WM) is a lymphoplasmacytic lymphoma, a malignant disorder that causes overproduction of monoclonal immunoglobulin M [2]. A common ocular symptom that occurs in WM patients is blurred vision due to retinal bleeding. Funduscopic examination in these patients commonly reveals distended and tortuous retinal veins and multiple flame-shaped or punctate retinal hemorrhages [3].

Cytomegalovirus (CMV) is a well-known cause of retinitis in immunosuppressed patients [2]. In addition, CMV is an infrequent cause of acute retinal necrosis (ARN), which is a rare cause of blindness in healthy adults [4]. Patients typically present with vitritis, occlusive vasculitis, and peripheral retinal necrosis, with an average duration of symptoms of 15 days [5]. Rhegmatogenous retinal detachment (RRD) is a common complication of ARN, occurring in 50%-75% of eyes and manifesting one to two months after the onset of retinitis [6].

We report a case of a patient with WM and CMV-related ARN who developed tractional retinal detachment (TRD) four years after the diagnosis and successful treatment of ARN syndrome.

## Case presentation

An 86-year-old male was referred to our vitreoretinal clinic, with reduction of vision in the right eye for 24 hours. His medical history included treated WM. His family history was unremarkable. His ocular history included a previous vitrectomy in the same eye four years ago for polymerase chain reaction confirmed CMV-related ARN. At that point, the patient was treated with two intravitreal injections of ganciclovir followed by oral ganciclovir for three months. His visual acuity was 10/10 in both eyes until presentation.

**Figure 1:**
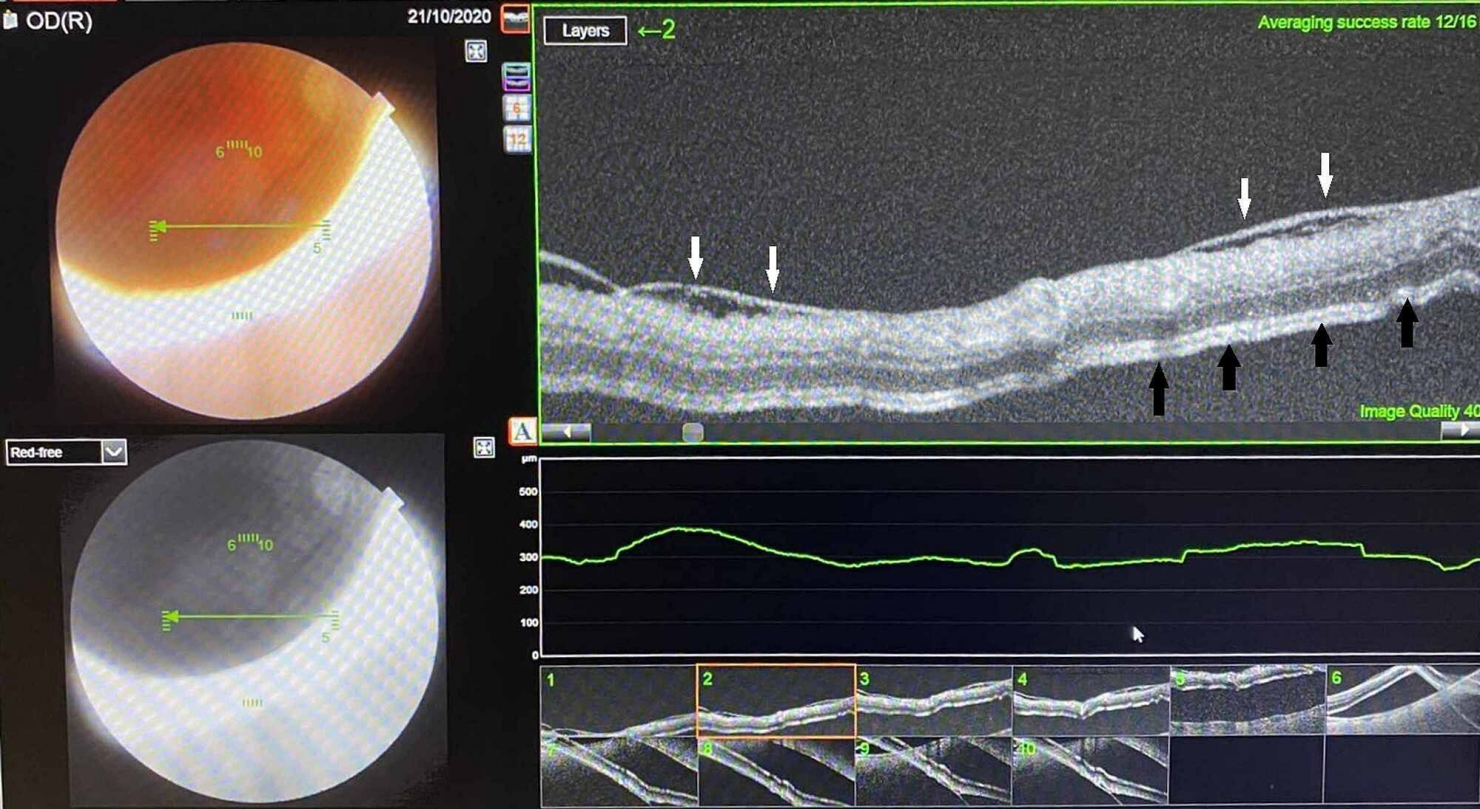
Optical coherence tomographic image demonstrating the epiretinal membrane (white arrows) and the tractional retinal detachment (black arrows).

Best corrected distance visual acuity was 4/10 in the right eye and 10/10 in the left eye (Snellen chart). Anterior segment examination was unremarkable, and fundus examination revealed a tractional macula-off retinal detachment (RD) (Figure 1).

The patient underwent 25G three-port pars plana vitrectomy with extensive epiretinal membrane (ERM) peeling and 20% sulfur hexafluoride gas injection (although a retinal break was not found, gas was used in order to facilitate subretinal fluid absorption). Three months postoperatively, the retina was flat and the visual acuity improved to 8/10.

## Discussion

ARN is a rare cause of vision loss in adults, which is caused by human herpes viruses (varicella-zoster virus, herpes simplex virus types 1 and 2, and rarely CMV and Epstein-Barr virus) [6]. It can affect immunocompetent or immunosuppressed patients of either gender at any age. In 1971, Urayama et al. first recognized a syndrome of acute panuveitis with retinal periarteritis progressing to diffuse necrotizing retinitis and RD [6].

The presenting symptoms are blurry vision, irritation, redness, photophobia, periorbital pain, and floaters in the affected eye. Funduscopy reveals focal and well-demarcated areas of rapid retinal necrosis of the peripheral retina, evidence of occlusive vasculopathy, and inflammatory reaction in the vitreous and anterior chamber episcleritis, scleritis, or keratic precipitates [6]. Optic nerve involvement is characterized by optic neuritis and limits the final visual potential [7].

Complications include cystoid macular edema, reproliferation, and RRD, which is a frequent complication with a poor prognosis. In the acute phase of ARN, there is an increase in vitreous turbidity due to the massive breakdown of the blood-ocular barrier, which leads to the formation of membranes and the development of severe proliferative vitreoretinopathy [7]. RRD with ARN occurs in more than 50% of eyes, due to severe retinal ischemia and vitreous organization. It is a serious late complication, which manifests one to two months after the onset of retinitis [6,8] and usually results in severe visual loss. RD usually develops as a result of retinal breaks located at the junction of the involved and uninvolved retina. These breaks are often located posteriorly and may be quite large. Both situations may create complex combined tractional and rhegmatogenous RD [9].

Prophylactic laser photocoagulation and prophylactic vitrectomy were proposed as two methods for preventing RD in ARN syndrome. Laser photocoagulation is aimed at reducing the risk of RRD by inducing retinal scarring at the central border of the necrotic retina [5]. This method still remains controversial as a way to prevent RD, and its use is limited by vitreous opacity and other complications [7]. Vitrectomy may help eliminate inflammatory factors, prevent lesions from occurring and extending, relieve vitreoretinal traction, and prevent RRD. Prophylactic vitrectomy significantly improves the anatomical and visual prognoses and seems to prevent RRD in cases with ARN [10].

Our patient developed ERM and TRD four years after the diagnosis and treatment of ARN syndrome, for which we performed a prophylactic vitrectomy. To our knowledge, no similar case of ARN and late onset of TRD after prophylactic vitrectomy has ever been mentioned in the literature. More studies are necessary to elucidate the possible mechanism of traction caused by the remaining peripheral vitreous after prophylactic vitrectomy in eyes with ARN syndrome.

## Conclusions

ARN syndrome is a rare cause of vision loss in adults, which is caused by human herpes viruses. It presents with blurry vision, irritation, redness, photophobia, periorbital pain, and floaters in the affected eye. RD is a serious complication, which occurs in more than 50% of eyes, manifesting one to two months after the onset of retinitis and usually resulting in severe visual loss. A late onset of TRD, four years after the diagnosis and treatment of ARN syndrome, is possible even after prophylactic vitrectomy.
